# Validation of the Global Treatment Burden Question (GTBQ): A novel single-item measure for use in clinical and research settings

**DOI:** 10.1177/26335565261443779

**Published:** 2026-05-02

**Authors:** Chloe Gamlin, Rachel Johnson, Lauren J. Scott, Anastasiia Kovalenko, Rebecca Goulding, Thomas Brain, Simon Chilcott, Thomas Blakeman, Jose M. Valderas, Simon D. S. Fraser, Polly Duncan

**Affiliations:** 1 Centre for Academic Primary Care, 1980 University of Bristol, Bristol, UK; 2 National Institute for Health Research Applied Research Collaboration West (NIHR ARC West), University Hospitals Bristol and Weston NHS Foundation Trust, Bristol, UK; 3 Population Health Sciences, Bristol Medical School, 1980 University of Bristol, Bristol, UK; 4 Division of Musculoskeletal and Dermatological Sciences, School of Biological Sciences, Faculty of Biology Medicine and Health, 5292 The University of Manchester, Manchester, UK; 5 Centre for Primary Care and Health Services Research, 5292 The University of Manchester, Manchester, UK; 6 Exeter Collaboration for Academic Primary Care, University of Exeter, Exeter, UK; 7 Yong Loo Lin School of Medicine, National University of Singapore, Singapore; 8 School of Primary Care, Population Sciences and Medical Education, Faculty of Medicine, 12211 University of Southampton, Southampton, UK

**Keywords:** multimorbidity, treatment burden, validation

## Abstract

**Background:**

A single-item, global measure could be valuable for identifying patients with high treatment burden within clinical practice and research.

**Aim:**

To validate a novel single-item global measure, named the ‘Global Treatment Burden Question’ (GTBQ).

**Methods:**

GTBQ: “how hard have you found the work of looking after your health conditions?” (responses “not hard”, “slightly hard”, “moderately hard”, “very hard”, “extremely hard”). Included participants: 18-65 years, ≥2 long-term conditions. Baseline survey: GTBQ, socio-demographics, Multimorbidity Treatment Burden Questionnaire (MTBQ), quality of life, health literacy. Follow-up survey: GTBQ. Electronic health records data: long-term conditions, consultations. Spearman’s Rank correlation (R_s_) and intraclass correlation coefficient (ICC) assessed construct validity and test-retest reliability. GTBQ performance was examined against global MTBQ scores to determine optimal thresholds.

**Results:**

974 (mean age 51) and 97 participants returned baseline and follow-up surveys, respectively. Responses were positively skewed with 22% reporting no burden at baseline. GTBQ scores were positively associated with global MTBQ scores (R_s_ 0.70); weakly associated with health literacy (R_s_ 0.36) and healthcare use (R_s_ 0.27); and negatively associated with physical health (R_s_ –0.66) and mental health (R_s_ –0.65). ICC was 0.66. GTBQ demonstrated excellent ability to discriminate between high and non-high treatment burden (area under the curve 0.838). Applying a GTBQ threshold of ≥3 yielded specificity 90%, sensitivity 53%, positive-predictive-value 82%, and negative-predictive-value 69%, indicating utility in ‘ruling in’ high treatment burden.

**Conclusion:**

This novel single-item measure has demonstrated good content and construct validity, moderate test-retest reliability, and strong ability to discriminate between high and non-high treatment burden.

## Introduction

Treatment burden is the effort required of patients to look after their health and the impact this has on their everyday life.^1^ This includes taking medication, monitoring health conditions (e.g. blood pressure or glucose monitoring), co-ordinating and attending appointments in different healthcare settings, arranging time off work to attend appointments and interacting with a range of healthcare professionals. High treatment burden is associated with poor health outcomes, including reduced quality of life and decreased concordance with medical treatment.^2–4^

Multimorbidity (multiple long-term conditions in the same individual) is common, with up to two-thirds of patients consulting in UK general practice having two or more long-term conditions.^5^ High treatment burden is associated with increased number of long-term conditions.^2,3,6,7^ Healthcare services are often organised around specific disease areas rather than individual patients. People with multimorbidity are therefore required to attend separate appointments and often need multiple medical treatments to manage their cluster of conditions.

Patient reported outcome measures (PROMs) have been developed and validated to measure treatment burden in research settings.^2,8–10^ Short measures of treatment burden for use in everyday clinical practice are lacking, however. We are aware of several existing PROMs (the Patient Experience with Treatment and Self-management pilot tool (PETS-Now), the Multimorbidity Treatment Burden Questionnaire (MTBQ;10 and 13 item versions), the Instrument for Patient Capacity Assessment (ICAN) Discussion Aid, and the Morris Single-item Measure).^2,11–14^ These PROMS have the potential to be used in a clinical setting, but all have limitations.

The Patient Experience with Treatment and Self-management PETS-Now was developed in the United States (US) from the original PETS questionnaire (60-item) and Brief PETS (32-item) as a novel, computer-based tool for use in clinical practice.^11,15,16^ The tool was designed to be completed in a clinical setting using a computer tablet. The patient selects a single treatment burden domain they are finding most difficult from a list of eight domains (e.g. monitoring health, medicine, getting health info etc) and then answers questions related to that domain. PETS-Now was co-designed by patients, clinicians and academics, and was found to be acceptable.^11^ Limitations of the tool are that patients can only report on one aspect of treatment burden, and it does not include a global treatment burden measure.

The Multimorbidity Treatment Burden Questionnaire (MTBQ) is a 10-item measure of treatment burden developed and validated in the UK.^2,13^ There is also an extended, 13-item version of the MTBQ which includes a further three optional questions. The questionnaire includes a range of treatment burden domains and produces a global score. This PROM demonstrated good content validity, construct validity, internal consistency reliability and responsiveness in the research setting. However, the questionnaire has not been validated for use in a clinical setting.

The ICAN tool was developed to aid discussion between patients and clinicians about the burden of looking after their health and their capacity to do this.^12,17^ Patients complete three questions: first, a checklist asking ‘are these areas of your life a source of satisfaction, burden, or both?’, second, an open ended question ‘what are the things that your doctors or clinic have asked you to do to care for your health... do you feel that they are a help, a burden, or both?’, and third, a space for free text comments. The clinician then uses one of three set opening questions to spark conversation based on the questionnaire responses, and asks in more detail ‘what stands out to you on this sheet you filled?’ The discussion aid was designed to support relationships between patients and their healthcare team, however, it has not undergone further testing or development for general clinical use.

In the UK, Morris et al. explored a novel single-item measure of treatment burden: ‘On a scale of 0–10, where 0 is no effort and 10 is the highest effort you can imagine, how would you rate the amount of effort you have to put in to manage your health conditions?’^14^ Study findings suggested the tool may have some utility in ruling out high treatment burden. While novel, the measure was not subject to any formal development processes and the authors described it as a ‘starting point’ for iterative work with a patient group.

We aimed to validate a novel single-item global measure of treatment burden, named the ‘Global Treatment Burden Question’ (GTBQ).

## Methods

### Study setting & design

The GTBQ was developed and validated as part of the ‘Supporting People to Live Well with Multiple Long-Term Conditions’ (SPELL) study, a multi-centre mixed methods study of treatment burden for adults aged 18 to 65 years with multimorbidity in the UK.^18^ A key aim of the wider SPELL study was to explore treatment burden in younger adults (18-65 years) with multimorbidity – an under researched group. We performed a cross-sectional study to validate the GBTQ.

### GTBQ development

The initial GTBQ was based on the validated MTBQ and the concept of a self-rated global score from the single-item measure.^2,13,14^ The GTBQ was designed with ease of patient and healthcare provider use in mind, intending to produce a validated global score and to generate discussion between patients and clinicians. Development included three rounds of cognitive interviews with adults with multimorbidity (15 interviews in total), with the GTBQ being refined after each round of interviews. The outcome of this iterative process was the GBTQ; a single question to screen for high treatment burden. A detailed account of the GBTQ development has been reported separately Ref to Development Paper.

### Structure and content of GTBQ

The GTBQ comprises a short explanation of the concept of treatment burden and a single global rating question, “Thinking about the last three months, how hard have you found the work of looking after your health conditions?” Response options are “not hard”, “slightly hard”, “moderately hard”, “very hard” and “extremely hard”. The GTBQ, was developed as part of a PROM called the ‘Short Treatment Burden Questionnaire’ (STBQ). This includes the GTBQ (section 1) and two additional sections. Section 2: “Please tick any things you have found hard in the last three months” (list of 13 options). Section 3: free text question, “Is there anything you want to tell us about the things you find hard?” This paper focuses on validating section 1 (the GTBQ).

### Study population, eligibility criteria and recruitment

Participants aged 18-65 with two or more long-term conditions (included in the Cambridge Multimorbidity Score) were recruited from 20 general practices serving a range of populations (e.g. socioeconomically deprived/affluent, rural/urban) in and around Bristol and Greater Manchester, UK.^19^ Practices were sampled to ensure that areas of socio-economic disadvantage were represented, and more participants were invited from practices serving deprived populations (Index of Multiple Deprivation (IMD) deciles 1-5).^20^ Staff from participating general practices identified potential participants through a pre-prepared search of the electronic health records (EHR). . Patients with dementia, those lacking capacity to consent, patients receiving palliative care, and nursing or care home residents were excluded.

Participants were offered a choice of online or postal survey for both the baseline and follow up surveys. Initially, patient participants were sent a paper invitation letter with study information. A paper copy of the questionnaire was enclosed, along with a QR code link to an online version for participants to complete according to their preference. One reminder was sent to non-responders by post, email, text or telephone. For the follow up questionnaire, an additional consent form was sent to participants alongside the baseline questionnaire, and participants were given a choice of online or paper versions.

A follow up survey was sent to a subsample of 180 participants from five of the original 20 practices, 1-4 weeks after their baseline surveys were returned. The responses generated from this subsample were used to measure test-retest reliability of the GTBQ. Participants were sent a £5 Love2Shop voucher for each completed questionnaire. Data collection for baseline and follow-up surveys was undertaken between March and July 2024.

### Survey content

The baseline survey included socio-demographic information (age, gender, ethnicity, employment status and postcode); the STBQ (including the GTBQ), the 13-item MTBQ (comparator measure of treatment burden^2,13^; the Patient-Reported Outcomes Measurement Information System (PROMIS-10) measures of physical and mental health related quality of life^21^; and the Single-item Literacy Screener (SILS) to detect limited health literacy).^22^ The follow-up survey included only the STBQ.

### Data from electronic GP records

Anonymised data were obtained from the electronic health records (EHR) of consenting participants, including age, sex, long-term conditions (from a list of 20 included in the Cambridge Multimorbidity Score; CMS)^19^ and number of general practice consultations recorded in the preceding 12 months.

Multimorbidity was measured using the validated 20-item CMS.^19^ For each participant, a score was assigned based on the presence of the condition: 1 for present and 0 for absent. Each condition was then assigned a weight using the ‘general outcome’ weighting. For instance, the weight for anxiety/depression (0.47) was multiplied by 1 if the condition was present. A CMS score was calculated for each participant by adding up the weights of their conditions.

### Analysis

The analysis was performed in Stata (version 18).^23^ Descriptive statistics were used to summarise participant characteristics. Psychometric properties of the GTBQ were tested against the International Society for Quality of Life Research (ISOQOL) standards.^24^ The analysis plan and results are reported in reference to the six ISOQOL recommended standards:

#### Conceptual and measurement model

##### Conceptual framework

Please see the development of the questionnaire section.

##### Question properties

Question properties were assessed by examining the distribution of responses to the GTBQ, and the proportion of missing responses at baseline.

##### Dimensionality

Not applicable as the GTBQ is a single-item PROM.

#### Reliability

To assess test–retest reliability, we calculated the intraclass correlation coefficient (ICC) to assess the agreement (along with the 95% confidence interval) between the GTBQ scores at baseline and follow-up.^25^ Values of <0.50, 0.50-0.74,0.75-0.89, and ≥0.90 indicate poor, moderate, good, and excellent reliability, respectively.^25^ Follow-up surveys completed within six weeks of the baseline survey were included in the analysis.

#### Validity

##### Content validity

The content validity of the GTBQ was assessed via 15 cognitive interviews in the iterative development stage of constructing the questionnaire. A detailed account of the GTBQ development has been reported separately.^26^

##### Construct validity

A GTBQ global score was generated by assigning a numerical value between 0 to 4 to the response selected by the study participant. Scoring was as follows: 0 “not hard”, 1 “slightly hard”, 2 “moderately hard”, 3 “very hard” and 4 “extremely hard”.

Construct validity was examined by testing five pre-specified hypotheses: first, a positive association between GTBQ and MTBQ scores^2,13^; second, a positive association between GTBQ score and number of general practice appointments in the preceding 12 months; third, a negative association between GTBQ score and health literacy (SILS)^22^; fourth, a negative association between GTBQ score and physical health (PROMIS-10 Global Physical Health)^21^; and fifth, a negative association between GTBQ score and mental health (PROMIS-10 Global Mental Health).^21^ To test these hypotheses, we applied Spearman’s rank correlation (R_s_).

##### Responsiveness

Assessing responsiveness was beyond the scope of this study.

#### Interpretability and test performance

To assess the interpretability of the GTBQ, the sensitivity, specificity, positive predictive value, and negative predictive value at each score were calculated. A global MTBQ score of ≥22 was used as a reference standard for high treatment burden.^2^ A receiver operating characteristic (ROC) curve was generated, and the area under the curve (AUC) was calculated to assess the effectiveness of the GTBQ at discriminating between high and non-high treatment burden.

We dichotomized the global score into non-high treatment burden (GTBQ 0-2) and high treatment burden (GTBQ 3-4). This binary enables clear identification of those who have high treatment burden compared to those who do not. We then summarized the participant characteristics and key outcome variables, including number of long-term conditions, across these two categories.

#### Translation

The GBTQ was only administered in English in this study.

#### Demands on patient respondents and investigators

Demands on patient respondents were assessed during the cognitive interviews. Demands on investigators were not formally assessed given the minimal response processing required to generate a global score.

### Sample size

Sample size calculations were conducted to ensure that the assessment of test-retest reliability would yield an interval estimate with adequate precision, rather than aiming for a specific hypothesis test power.^27^ To achieve a 95% confidence interval (CI) with a width of 0.2 (i.e., ranging from 0.6 to 0.8) for an ICC of 0.7, 101 participants were needed to complete both the baseline and follow-up questionnaires.

Power calculations were performed to determine a baseline questionnaire sample size for the original SPELL study. This was an exploratory study without one specific hypothesis. The calculations were based on a total sample size of 1,000, a significance level of 0.05, and a binary exposure where the prevalence of that exposure was allowed to vary from 0.2 to 0.5. The baseline risk of high burden was 20% with a risk of high burden of 30% in the group with the exposure. This gave an observed risk of high burden in the population close to what was reported in the original MTBQ paper (26.6%).

### Patient and public involvement (PPI)

A PPI group, consisting of eight people with lived experience of multimorbidity, was established at the outset of the SPELL study. The members of this group contributed to the development of the research questions, design of the study and creation of study documents including the initial, interim and final versions of the GTBQ.

### Ethical approval and data sharing

Participants gave informed consent to participate in the study before taking part. The SPELL study has ethical approval from the London – Westminster Research Ethics Committee (REC reference 22/PR/1750). Finalised, tabulated data will be freely available as a Final Study Report from the University of Bristol Research Portal.

## Results

8532 eligible patients were invited to participate in the baseline survey, resulting in 974 participants (11% response rate) included in the analysis, of whom 968 completed the global GTBQ. Of these, 180 individuals were invited to complete the follow-up survey. 107 completed follow-up surveys were returned, with 97 included in final analysis after removing duplicate responses.

### Participant characteristics

The mean age of the 974 baseline survey participants was 51 years (SD 11), with most (64%) between 50-65 years; 89% of respondents were white and 62% were female (Table 1). There was higher representation of participants from more deprived areas (42% in IMD quintile 1 and 19% in IMD quintile 2). A third of participants were in full time employment and a further 21% employed part time. The most common long-term conditions were anxiety and/or depression (62%), hypertension (38%), painful conditions (35%) and asthma (28%). The 97 participants in the follow up survey were a subsample of the 974 baseline survey participants. Compared to the whole sample, the follow up sample group characteristics were similar. Follow up survey participants had a mean age of 51 years (SD 11), 63% between 50-65 years, 91% were white, 62% were female and 56% were in employment. Again the most common long-term conditions were anxiety and/or depression (62%), hypertension (47%), painful conditions (32%) and asthma (32%).

**Table 1.** Baseline participant characteristics and GTBQ scores.

​	All participants n (%)	Participants with follow up data n (%)
Total	974	97
Age (mean, SD) [missing n = 41, 4%]	51 (11)	51 (11)
18-29	57 (6%)	5 (5%)
30-39	120 (12%)	12 (12%)
40-49	174 (18%)	19 (20%)
50-59	326 (34%)	30 (31%)
60-65	295 (30%)	31 (32%)
Gender [Missing n = 0]		
Male	361 (37%)	35 (36%)
Female	604 (62%)	60 (62%)
Other	9 (1%)	2 (2%)
Ethnicity [Missing n =0]		
White	868 (89%)	88 (91%)
Asian/Asian British	44 (5%)	4 (4%)
Black/African/Caribbean/Black British	31 (3%)	1 (1%)
Any other ethnic group	31 (3%)	4 (4%)
Employment status [Categories not mutually exclusive, missing n = 15, 1.5%]		
Carer	68 (7%)	8 (8%)
Employed	492 (50%)	54 (56%)
Self employed	54 (6%)	8 (8%)
Student	22 (2%)	0 (0%)
Retired	100 (10%)	17 (18%)
Permanently sick/disabled	191 (20%)	15 (15%)
Unemployed	109 (11%)	7 (7%)
Other	18 (2%)	0 (0%)
Index of multiple deprivation quintile [Missing n = 43, 4.4%]		
1 (most deprived)	395 (42%)	29 (32%)
2	181 (19%)	22 (24%)
3	113 (12%)	19 (21%)
4	134 (14%)	18 (20%)
5 (least deprived)	108 (12%)	4 (4%)
Number of long-term conditions* [Missing n = 0]		
0-2	462 (47%)	49 (51%)
3-4	385 (40%)	37 (38%)
≥5	127 (13%)	11 (11%)
Long term condition* [Missing n = 0]		
Alcohol	120 (12%)	6 (6%)
Hypertension	373 (38%)	46 (47%)
Hearing Loss	182 (19%)	18 (19%)
Diabetes	216 (22%)	19 (20%)
IHD	67 (7%)	4 (4%)
CKD	55 (6%)	4 (4%)
AF	34 (3%)	4 (4%)
Constipation	61 (6%)	5 (5%)
COPD	52 (5%)	6 (6%)
CTD	107 (11%)	14 (14%)
Cancer	57 (6%)	7 (7%)
Heart Failure	15 (2%)	1 (1%)
Psychosis	59 (6%)	7 (7%)
Anxiety/Depression	606 (62%)	60 (62%)
IBS	233 (24%)	16 (16%)
Asthma	270 (28%)	31 (32%)
Epilepsy	22 (2%)	2 (2%)
Stroke/TIA	51 (5%)	2 (2%)
Pain	339 (35%)	31 (32%)
GTBQ score [Missing n = 6, 0.6%]		
0 (no burden)	217 (22%)	17 (18%)
1 (low burden)	223 (23%)	27 (28%)
2 (moderate burden)	244 (25%)	27 (28%)
3 (high burden)	187 (19%)	20 (21%)
4 (very high burden)	97 (10%)	6 (6%)

*Most common 20 long-term conditions included in the Cambridge Multimorbidity Score^19^.

#### Conceptual and measurement model

##### Conceptual framework

The GTBQ was adapted from the original validated MTBQ.^2,13^ The focus of this paper is to validate the GTBQ global rating score. The STBQ was developed with three rounds of five cognitive interviews comprising a think-aloud task and prompts leading to modifications of the STBQ at each round. The GBTQ is a single item question designed to screen for high treatment burden, and a detailed description of the development of the GTBQ and wider STBQ has been reported separately.^26^

##### Question properties

The GTBQ was completed by 968 SPELL study participants (99.4%). The GTBQ scores were positively skewed, with 22% of participants reporting no burden (GTBQ score 0) at baseline.

##### Dimensionality

Not applicable.

#### Reliability

The ICC for agreement between GTBQ scores at baseline and follow-up was 0.66 (95% CI 0.53 - 0.76). This indicates moderate test-retest reliability.^25^

#### Validity

##### Content validity

**Figure 1.**
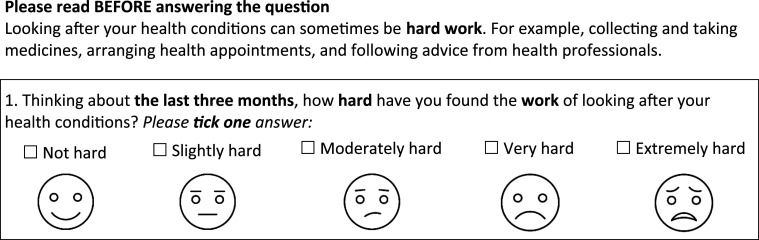
Global Treatment Burden Question (GTBQ) final version.

**Figure 2.**
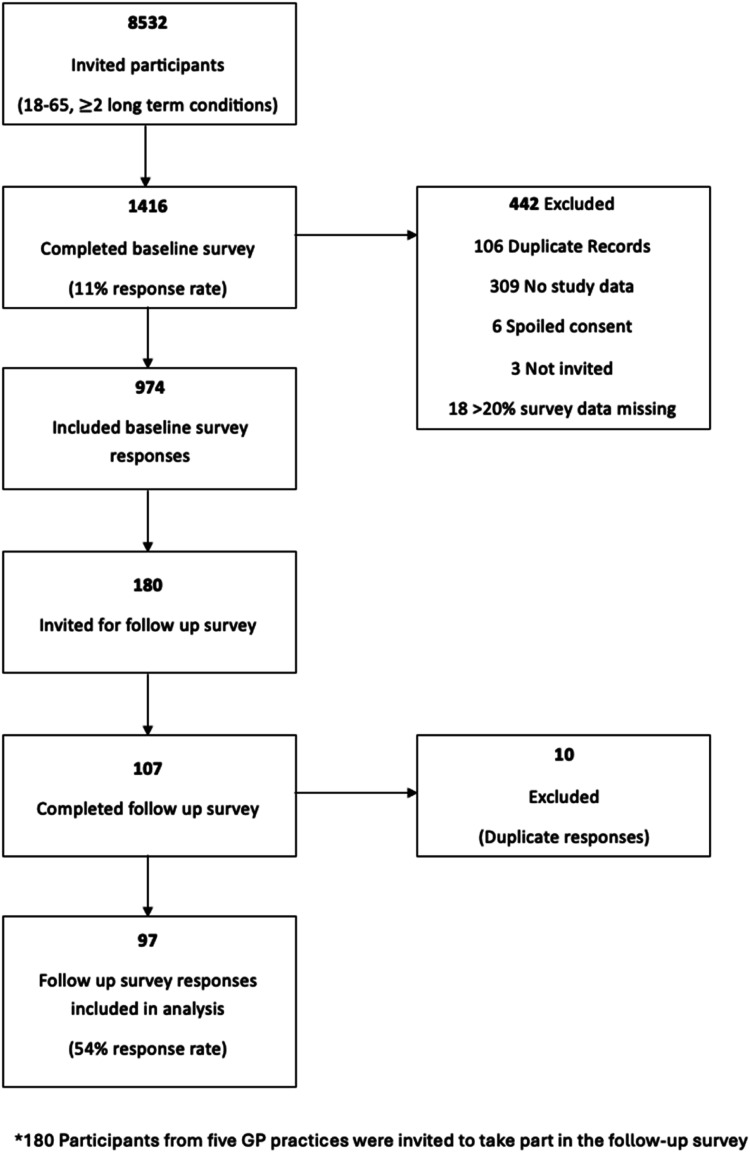
Participant flow diagram.

A detailed account of the GTBQ development has been reported separately.^26^ The STBQ underwent iterative development in response to discussion with the PPI group and through cognitive interviews. The 5-point Likert scale for the GBTQ was discussed with the wider research team and PPI group after issues were identified during the cognitive interviews. The response options were revised for clarity (Figures 1 and 2). As an example, the response option “a little” was changed to “slightly” while “quite” became “moderately”.

##### Construct validity

As hypothesised, there was a moderately strong positive association between the GTBQ score and comparator global MTBQ score (10-item MTBQ r_s_ 0.71, 13-item MTBQ 0.70) (Table 2).^2^ There were also weak positive associations between GTBQ score and healthcare use (r_s_ 0.27), and GTBQ score and lower health literacy (SILS questionnaire; r_s_ 0.36).^22^ There were negative associations between the GTBQ score and PROMIS-10 global physical health (r_s_ -0.65) and PROMIS-10 global mental health scores (r_s_ -0.66).^21^ These findings provide evidence of construct validity of the GTBQ score.

**Table 2.** Correlations between GTBQ score and global MTBQ score, health literacy, healthcare use and health related quality of life.

Variable	Spearman’s rank correlations (r_s_)	P values
MTBQ (10 item)	0.71	<0.0001
MTBQ (13 item)	0.70	<0.0001
SILS	0.36	<0.0001
Healthcare use (GP consultations)	0.27	<0.0001
PROMIS-10 global mental health	-0.66	<0.0001
PROMIS-10 global physical health	-0.65	<0.0001

##### Responsiveness

Not applicable.

#### Interpretability and test performance

**Figure 3.**
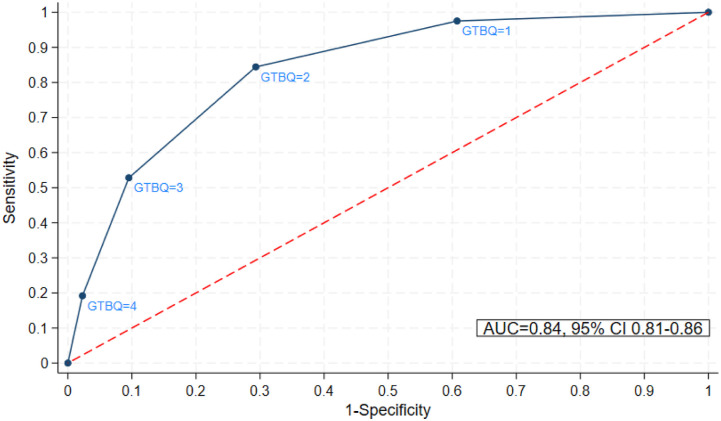
Receiver operating characteristic curve for the GBTQ.

The receiver operating characteristic (ROC) curve is shown in Figure 3. The area under the curve was 0.838 (95% CI =0.814-0.862), indicating excellent ability to discriminate between high and non-high treatment burden. Applying a GTBQ threshold of ≥3 for high treatment burden yielded a specificity of 90%, sensitivity of 58%, positive predictive value of 82% and negative predictive value of 69% (Table 3). Setting a GTBQ threshold of ≥2 yielded a specificity of 71%, sensitivity of 84%, positive predictive value of 71% and negative predictive value of 84%.

**Table 3.** Sensitivity, specificity, predictive values and likelihood ratios of GTBQ scores to predict high treatment burden (a global score of ≥22 on the 13-item MTBQ).

GTBQ score	Sensitivity	Specificity	Positive predictive value	Negative predictive value	Positive likelihood ratio	Negative likelihood ratio
0	1	0	​	​	​	​
1	0.98	0.39	0.58	0.95	1.60	0.06
2	0.84	0.71	0.71	0.84	2.88	0.22
3	0.53	0.90	0.82	0.69	5.55	0.52
4	0.19	0.98	0.88	0.59	8.39	0.83

Descriptive data suggested possible differences in participant characteristics across the two treatment burden score groups (non-high treatment burden and high treatment burden using the threshold of GTBQ score ≥3) (Table 4). People who were of a younger age, female gender, or living in more deprived areas appeared to be more likely to report high treatment burden. Furthermore, our descriptive data highlights the possibility of higher treatment burden reported by participants with psychosis, painful conditions, constipation and epilepsy, and those with a higher Cambridge multimorbidity score (CMS).^19^ We did not test for significance or adjust for confounding variables as these findings are being reported elsewhere.

**Table 4.** Comparison of characteristics between participants with non-high and high treatment burden.

​	Non-high treatment burden (GTBQ=0-2)	High treatment burden (GTBQ=3-4)
	n (%)	n (%)
Total	684	284
Age (mean, SD)	52.1 (11.2)	48.5 (11.8)
Gender		
Male	276 (40%)	85 (20%)
Female	406 (59%)	192 (68%)
Ethnicity		
White	610 (89%)	253 (89%)
Non-white	74 (11%)	31 (11%)
IMD quintile		
1 (most deprived)	251 (37%)	142 (50%)
2	134 (20%)	46 (16%)
3	75 (11%)	38 (13%)
4	101 (15%)	30 (11%)
5 (least deprived)	89 (13%)	19 (7%)
Cambridge Multimorbidity Score (mean, SD)	1.35 (0.92)	1.68 (0.96)
Long term conditions		
Alcohol	74 (11%)	46 (16%)
Hypertension	291 (43%)	80 (28%)
Hearing Loss	129 (19%)	50 (18%)
Diabetes	158 (23%)	57 (8%)
IHD	55 (8%)	12 (4%)
CKD	38 (6%)	17 (6%)
AF	31 (5%)	3 (1%)
Constipation	34 (5%)	25 (9%)
COPD	32 (5%)	20 (7%)
CTD	65 (10%)	39 (14%)
Cancer	46 (7%)	11 (4%)
Heart Failure	10 (15%)	5 (2%)
Psychosis	29 (4%)	28 (10%)
Anxiety/Depression	373 (55%)	227 (80%)
IBS	150 (22%)	81 (29%)
Asthma	189 (28%)	80 (28%)
Epilepsy	13 (2%)	9 (3%)
Stroke/TIA	42 (6%)	9 (3%)
Pain	192 (28%)	144 (51%)

#### Translation

Not applicable.

#### Demands on patient respondents and investigators

The GTBQ is a single-item global measure of treatment burden. Response options for the global question (GBTQ) were presented in both words and pictures (smiley faces). Missing data were minimal with 0.6% baseline survey respondents not returning a completed baseline GTBQ score. Demands on investigators were also intended to be minimal, with no calculations required to generate a GTBQ score. The Short Treatment Burden Questionnaire (STBQ) comprises the GTBQ and two additional questions and fits onto a single A4 page.

## Discussion

### Summary of findings

We have validated a novel, single-item global measure of treatment burden, named the ‘Global Treatment Burden Question’ (GTBQ), designed for use in clinical practice and research. The psychometric properties of the GTBQ meet the standards set out by ISOQOL for a novel PROM, demonstrating good content validity, construct validity, interpretability and moderate test-retest reliability.^24^ Importantly, the GTBQ’s ability to discriminate between high and non-high treatment burden, based on the area under the curve, was excellent.

Applying a GTBQ threshold of ≥3 generated high specificity (90%) and positive predictive value (82%), indicating utility as a screening tool to identify patients with high treatment burden (low chance of false positives). Setting a GTBQ threshold of ≥3 yielded a moderate sensitivity (53%), indicating that some patients with high treatment burden would be missed (false negatives). The GTBQ is designed as a ‘rule in’ screening tool to detect high treatment burden. The implication of a sensitivity of 53% is that some patients with high treatment burden would go undetected and therefore not receive appropriate support. This could also lead to underestimation of the number of people with high treatment burden.

Applying a threshold of ≥2 improved sensitivity, but at the expense of poorer specificity, though the positive predictive value and negative predictive value remained high. Therefore, if using the GTBQ as a ‘rule in’ screening tool, we recommend applying a threshold of ≥3 to distinguish between high and non-high treatment burden. For simplicity, it is helpful to dichotomise the scale to identify those who have high treatment burden compared to those who do not. However, it may also be helpful to think of the five levels of the GTBQ as no burden, low burden, medium burden, high burden and very high burden.

Our descriptive analysis found that younger individuals living in more deprived areas with higher levels of multimorbidity (particularly painful or mental health conditions) were more likely to report high treatment burden. Further in-depth analysis of these data is planned (to be reported separately). These findings align with the existing literature. The association between younger age and high treatment burden is consistent across several studies using different PROMs.^2,3,7,14^ One explanation for this is that due to work and childcare commitments, younger adults may have less capacity to manage the work of looking after their health. The original MTBQ study did not find an association between high treatment burden and deprivation,^2^ but this has been reported in the US.^16^ Several studies using different PROMs report an association between high treatment burden and multimorbidity.^2,3,7,14^

A strength of this study is that we have validated the GTBQ in a large sample of individuals with multimorbidity. Our sample included a high proportion of individuals living in disadvantaged areas and a proportion of individuals from global majority ethnicities more closely reflective of the UK population than previous work in this area.^28^ This improves representation of a diverse population and subsequent generalisability. The GTBQ was developed using a robust process, involving cognitive interviews with people with multimorbidity. A key aim was for the PROM to be simply worded and user friendly. The full STBQ (including the GTBQ and two optional questions) fits on a single A4 page.

A limitation is that the tool has been validated only in adults aged 18-65. This age range was chosen deliberately due to limited research in younger adults with multimorbidity, the focus of the wider SPELL study.^18^ However, the GTBQ is designed to be used by adults of any age and it would be useful to validate it in older adults, as well as people with single long-term conditions. A further limitation is that we were unable to assess responsiveness to change. A final limitation is the low response rate. This may limit the generalisability of the study findings as those who took part may differ from those who did not take part. Other primary care and PROM validation studies have also reported low response rates of around 20-35%^13,16,29^ but not quite as low as this study (11%). One explanation is that response rates in the UK are known to be lower in individuals living in more disadvantaged areas^30^ and, to increase representation, we purposefully invited more people from practices serving disadvantaged populations.

### Conclusion

The GTBQ is a single-item, global measure of treatment burden that has demonstrated excellent ability to discriminate between high and non-high treatment burden. The brevity of the measure is a strength, particularly for use in clinical practice and trials with multiple outcome/*-s. The GTBQ was developed through a robust and iterative process and validated in a cross-sectional sample of adults with multimorbidity. It has demonstrated good content validity, construct validity, interpretability and moderate test-retest reliability. Further work is planned to assess the feasibility of using the GTBQ and STBQ (with two additional questions) in clinical practice.

## Data Availability

Access to anonymised data may be granted, where appropriate governance approvals are in place, following review via the University of Bristol Research Data repository https://data.bris.ac.uk/data/
